# Benefits of Metformin Repurposed for Alzheimer’s Disease Prevention, Evidence From Mendelian Randomization

**DOI:** 10.1093/geroni/igaf122.1578

**Published:** 2025-12-31

**Authors:** Jatupol Kositsawat

**Affiliations:** UConn Health, Farmington, Connecticut, United States

## Abstract

Previous observational studies reported a decreased incidence of dementia in type 2 diabetic patients who take metformin compared to other hypoglycemic drugs. The possible neuroprotective effects of metformin also showed dose-dependent effects, either the daily dosage or the accumulative dosages taken. However, the Mendelian randomization (MR) study, one of the causal inference methods robust to confounders and reverse causation, poses some challenges because no specific target genes are attributable to metformin. Instead, researchers have based their MR study on a downstream biomarker such as A1c. This talk will review current evidence of MR studies on metformin exposure that look through the mechanistic effects of metformin other than glycemic control reflected by A1c in more detail. A recent study showed evidence of metformin gene expression in reducing the risk of Alzheimer’s disease (AD) in the general population. Plausible mechanisms in AD protection include mitochondrial function and decreased mitochondrial complex 1-related gene expression. Another mechanistic link might be through a reduction in biological aging, an important process underlying AD pathogenesis. The MR studies of metformin effects showed benefits on phenotypic age and leukocyte telomere length. Our future goal is to thoroughly investigate the causal inference of metformin and other repurposed drugs for AD prevention through MR studies. A large number of observational study participants enable various sensitivity analyses that can show how these protective effects differ in different population characteristics and thus help identify patients who would benefit most from metformin following the Precision Gerontology concept.

